# A novel region within a conserved domain in ATG7 emerged in vertebrates

**DOI:** 10.1080/27694127.2022.2118933

**Published:** 2022-09-07

**Authors:** Valgerdur J. Hjaltalin, Vivian Pogenberg, Kevin Ostacolo, Arnar Pálsson, Margrét Helga Ogmundsdottir

**Affiliations:** a Department of Anatomy, Biomedical Center, Faculty of Medicine, University of Iceland, Sturlugata 8, 102, Reykjavik, Iceland; b Department of Biochemistry and Signal Transduction, University Medical Center Hamburg-Eppendorf (UKE), Hamburg, Germany; c Institute of Life and Environmental Sciences, University of Iceland, Sturlugata 7, 102 Reykjavik, Iceland

**Keywords:** ATG7, Autophagy, Evolution, Cancer, E1-enzymes

## Abstract

The E1-like enzyme ATG7 belongs to a group of ATG proteins that mediate the autophagy process. Autophagy is a highly conserved degradation pathway important for maintaining homeostasis in eukaryotic cells. Here, we study the evolution of E1 enzymes and specifically describe a region of ATG7 that emerged early in vertebrates. This vertebrate-specific region (VSR) is situated within the adenylation domain of the protein, which is the most conserved domain of E1 enzymes and is of prokaryotic origin. A comparative analysis revealed that ATG7 is unique in this respect, as in other E1 enzyme family members this domain is highly conserved from yeast to humans and has not experienced insertions of extra amino acids. The function of the VSR domain is unknown, but two residues within the region, D522 and S531 have been linked with cancer in humans. Analysis of natural selection indicates positive selection on S531 only on the mammalian clade. Notably, this was the only residue in ATG7 showing this signal. Interestingly, structural analysis of ATG7 predicted that the VSR may be intrinsically disordered and could harbor a macro-molecular binding site. Analysis of cells expressing ATG7 lacking the VSR indicated that these cells are unable to facilitate the lipidation of LC3, suggesting an important role of this region in autophagy.

**Abbreviations**: aBSREL - an adaptive branch-site random effects likelihood; AD - adenylation domain; ATGs - autophagy-related genes; Baf-A1 - Bafilomycin-A1; EV - empty-vector; CTD - C-terminal domain; ECTD - extreme C-terminal domain; EMT - epithelial-mesenchymal transition; FEL - fixed effects likelihood; GABARAP - gamma-aminobutyric acid receptor-associated protein; LC3 - microtubule-associated protein 1A/1B-light chain 3; MEFs - mouse embryonic fibroblasts; MOCS3 - molybdenum cofactor synthesis 3; NTD - N-terminal domain; UBL ubiquitin like protein; VSR - vertebrate specific region

## Introduction/background

Macroautophagy (hereafter referred to as autophagy) is a catabolic process where intracellular components, such as defective proteins or organelles, are degraded and either reused in anabolic reactions or exocytosed [1]. Autophagy occurs through the formation of a double- layered membrane, termed phagophore, which expands to engulf the cargo into a double-membraned vesicle, termed autophagosome. The autophagosome then proceeds to fuse with lysosomes to facilitate degradation [2]. This process is directed by a group of autophagy-related proteins (ATGs). Currently, over 40 *ATGs* have been identified, initially in yeast and subsequently in humans and various other eukaryotes. Of these, ⁓20 ATG proteins are estimated to represent the core autophagy machinery. Almost all the ATGs are evolutionary conserved though some components are present either in several copies or missing in specific taxa. Even some traces of distant ATG homologues exist in prokaryotes, such as cyanobacteria and euryarchaeota [3,4].

All higher eukaryotes, from yeast to humans, possess a full set of the core ATG proteins [3,4]. Evolutionary analyses show that gene duplications and losses have affected the autophagy system, both at the base of eukaryotes but also on specific branches. Some genes have been stable and were not retained in multiple copies after the Whole genome duplications at the base of vertebrates, such as ATG11 and ATG17. Others have expanded, perhaps most notably Atg8 into the LC3/GABARAPs which has occurred on multiple branches, including metazoans and plants [3,5]. Evolutionary studies indicate that the function of the ATGs participating in autophagy is highly conserved among eukaryotes, while species-specific adaptations of genes involved in selective autophagy are common, resulting in more divergent upstream signaling and regulation [6–8].

Each part of the autophagy process is influenced by a different complement of proteins. One of the best characterized steps is the elongation of the phagophore, which occurs through lipidation of LC3/GABARAPs (yeast Atg8). ATG7 is one of the core autophagy proteins and participates in two ubiquitin-like cascades that facilitate the lipidation of LC3/GABARAPs. Modifications of proteins by ubiquitin- and ubiquitin-like proteins is a three-step conjugation process catalyzed by members of the E1-, E2-, and E3 enzyme families. ATG7 belongs to the family of E1- and E1-like enzymes. Their role is to initiate the cascade by adenylation of a glycine residue at the C-terminus of the target protein [9–11]. Following activation, the E1-enzyme forms a covalent thioester bond which is then transferred over to an E2 enzyme. ATG7 activates two different ubiquitin-like proteins (UBL), ATG12 and LC3/GABARAPs, and pairs them with the E2 enzymes ATG10 and ATG3, respectively. ATG7 interacts with the E2 enzymes, ATG10 and ATG3, through the N-terminal domain [12]. Notably, all E1 enzymes have an adenylation domain that harbors the characteristic function of these ubiquitin-like proteins. The domain is of prokaryotic origin and generally considered to be conserved across all eukaryotes [10,13].

ATG7 is one of the most highly conserved ATGs with around 45% sequence identity between yeast and humans. It is homodimeric and mainly consists of two globular domains: An N-terminal domain (NTD) and a C-terminal domain (CTD) that are separated by a short flexible linker (see Figure 3A). Within the CTD resides the aforementioned adenylation domain (AD) and an extreme C-terminal ATG7-specific domain (ECTD). Unlike the canonical E1 enzymes, ATG7 lacks a separate catalytic cysteine domain, but instead contains a catalytic cysteine residue (C572) within the AD (Figure 3A). In addition, the NTD domain is unique to ATG7 and does not share significant homology with other E1 enzymes [12,14,15]. In humans, three protein-coding isoforms of ATG7 are known. Previously, we described an isoform of ATG7, ATG7(2), that is unable to lipidate LC3/GABARAPs, as it lacks 27 amino acids at the C-terminal necessary for binding this family of proteins. This isoform is therefore unable to carry out the characterized role of ATG7 in autophagy [16]. A few ATG7 variants have been linked to diseases in humans. Two missense mutations within the AD, V471A and D522E, which have been linked to earlier onset of Huntington disease and increased risk of hepatocellular- and cholangiocarcinoma in humans, respectively, and one nonsense mutation, R659* within the ECTD that was linked to familiar cholangiocarcinoma [17,18]. In addition, several mutations were detected within ATG7 of patients suffering from neurodevelopmental disorders, most are situated within the CTD [19].

Despite being primarily known as one of the key autophagy proteins it has become evident that ATG7 also plays autophagy independent roles in the cell. In fact, most of the key autophagy components have been shown to play other non-autophagy related roles [20–22]. The expression of ATG7 is frequently altered to affect autophagy function in experimental biology [23]. Therefore, it is important to the autophagy research community that other roles of ATG7, and other key autophagy proteins, are explored further. Alternative functions of ATG7 that have been described include regulating the transcription of cell cycle inhibitor p21^CDKN1A^ *via* binding of tumor suppressor p53; promoting E-cadherin expression, resulting in delayed epithelial-mesenchymal transition (EMT); and forming a complex with caspase-9, preventing apoptosis and instead leading to necrosis [24–28]. These studies and others have exclusively been performed in mammalian models. Therefore, it is unknown which of these functions ATG7 has in other taxa. Moreover, none of these novel functions have been assigned to a specific region of the protein. Knockout studies have shown that lack of ATG5 or ATG7 is neonatal lethal in mice, while fruit flies are viable [29,30]. This suggests a more important function of these proteins during development in mice and perhaps mammals or vertebrates. We set out to investigate the evolutionary history of ATG7 domains and functions. We wanted to know whether a new functional region had emerged in the history of eukaryotes that could harbor a novel function. The data indicates a novel protein region within the AD in ATG7 that emerged as recently as in vertebrates. This emergence is notable as the AD domain in other members of this protein family is generally highly conserved from yeast to humans. Interestingly, this novel region is predicted to be intrinsically disordered and a potential macro-molecular binding site. After this domain emerged it has remained relatively conserved within vertebrates. Analyses of mouse embryonic fibroblasts (MEFs) lacking the region indicate an important role in autophagy.

## Results

### A new region within the highly conserved adenylation domain of ATG7 emerged in vertebrates

**Figure 1.**
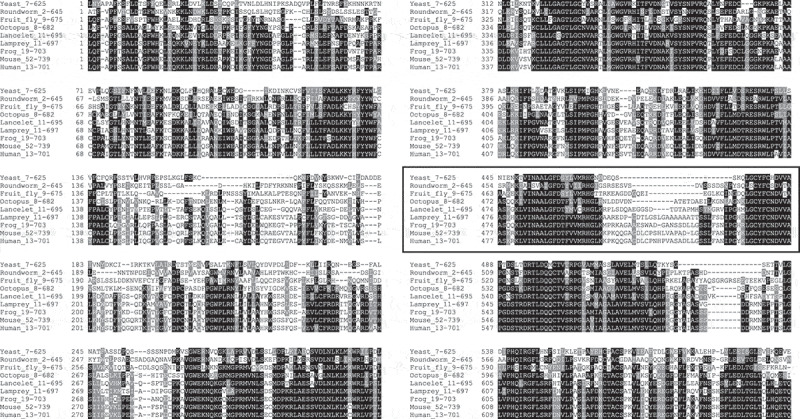
Multiple sequence alignment of ATG7 between distantly related species, revealing a region that appeared in early vertebrates (shown within a bold border). The ends have been trimmed to fit the columns. Which amino acids are shown from each species is indicated behind the species names. Generated with Clustal Omega.

ATG7 is present in all higher eukaryotes ranging from yeast to humans and is primarily known for its role in lipidation of the elongating autophagosomal membrane. However, a number of studies [24,27,31–36] have described autophagy-independent roles of the protein in mammals or vertebrates. To address whether these altered functions relate to a new protein region, that might harbor a new function, we searched Orthodb and NCBI for orthologues of this gene, initially in nine distantly related eukaryote species (human, mouse, frog, lamprey, lancelet, octopus, fruit fly, roundworm, and yeast). Multiple alignment revealed a region that emerged in early vertebrates (Figure 1, table S1 and supplemental figure S1 for comparison with another key autophagy gene, *ATG5*). The region, hereafter referred to as the vertebrate-specific region (VSR), is flanked by two highly conserved motifs within the AD. In yeast, the flanking regions join at position S479-S480, and are separated by extra amino acids, that tend to increase in number as the species are more closely related to vertebrates. The corresponding region in humans ranges from position L517-K545. Thus, this domain does not seem to have emerged in a single event, as there is progressive elongation of this region from more distant relatives to the most primitive vertebrates. While the alignment shows a gap of four amino acids in the lancelet, the chordate species often used as an outgroup in studies on novel vertebrate features [37], it is hard to conclude at which juncture in evolutionary history, the domain came into full form or function.

**Figure 2.**
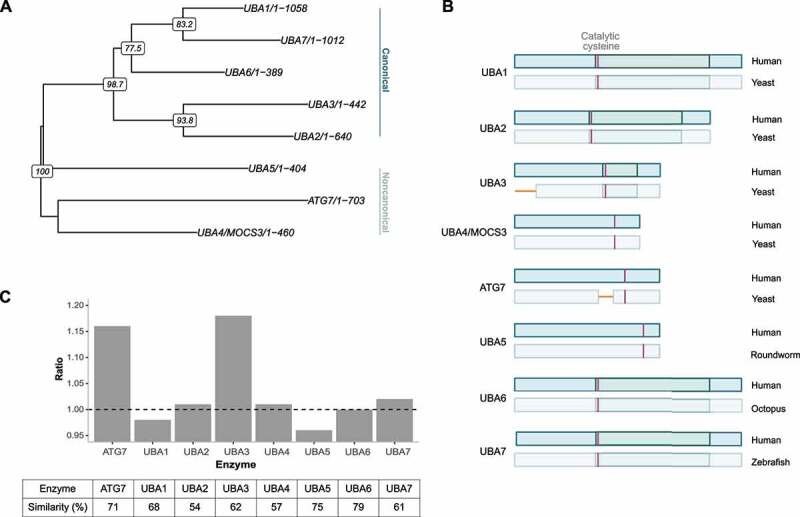
(**A**) Phylogenetic tree of E1- (canonical) and E1-like (noncanonical) enzymes that possess an active adenylation domain. ATG7 and UBA4/MOCS3 separated before LECA (last eukaryotic common ancestor). At LECA; three E1-enzyme families had emerged; UBA1-3. UBA5 emerged in protists and UBA6-7 emerged through gene duplications of UBA1 [11]. The tree was calculated with the R packages Ape and Phangorn, using neighbour joining, maximum likelihood and bootstrap (1000 replicates). The bootstrap values are shown at the nodes. (**B**) A comparison of the adenylation domain of E1- and E1-like enzymes (which possess an active adenylation domain) between humans and the deepest branch where each protein has been described. UBA5 emerged in protists and is not found in yeast. Neither are UBA6 or UBA7, which have only been described in higher eukaryotes. UBA3 and ATG7 contain gaps in yeast in comparison with humans (shown in yellow). Canonical E1-enzymes possess a specific catalytical cysteine domain within the AD (indicated with a darker shade of green) while noncanonical E1 enzymes only possess the catalytic cysteine residue (pink stripe). (**C**) Ratio of length of the AD where the same species were compared as in (**B**) for each E1 enzyme. Below are the percentage similarities between the same species. This shows that the AD is highly conserved in all enzymes, but the length of the AD region has increased in ATG7 and UBA3.

Notably, the VSR is situated between amino acids L517-K545 (based on the human protein) within the adenylation domain (AD). The AD is the most highly conserved part of E1-enzymes and is thought to predate the eukaryotes [13]. In order to evaluate the evolutionary stability of AD, and screen specifically for insertions and added protein domains, we conducted a comparative analysis of E1- and E1-like enzymes. Again, alignments revealed that the AD domain has generally remained the same from yeast to humans, in all other members of this enzyme family (Figure 2). Phylogenetic analysis of these E1 enzyme family members, considered to possess an active AD shows the first split within the E1 family occurred pre-LECA (last eukaryotic common ancestor) and gave rise to ATG7 and UBA4/MOCS3. At the time of LECA, three other UBA families had emerged; UBA1, UBA2, and UBA3. Divergence in protists gave rise to UBA5. UBA6 emerged in the common ancestor of fungi, slime molds and multicellular eukaryotes. Lastly, UBA7 emerged after gene duplication of UBA1 in vertebrates (Figure 2A) [11]. Despite long evolutionary history, indicated by deep splits within the E1 and E1-like family, the data indicate that the AD is highly conserved in all proteins (Figure 2B-C). A comparison between humans and the deepest branch where each protein has been described (yeast for UBA1-UBA4, roundworm for UBA5, octopus for UBA6 and zebrafish for UBA7) shows that the amino acid similarity percentage range from 54-79%. Notably, no new regions have emerged within the AD domain in any of these genes, exception being the VSR of ATG7 and a region at the beginning of the AD domain of UBA3, that is absent in yeast but has emerged in roundworm. An alignment of UBA3, between 15 species from yeast to humans, indicated that the gap is specific to *S. cerevisiae* as the same region in *Schizosaccharomyces pombe* (*S. pombe*) was comparable to that of higher eukaryotes (figure S2). In this study we focus on the VSR of ATG7.

We emphasize that while the VSR emerged in vertebrates, the AD remains highly conserved. This implies that the addition of this new domain has not interfered with the structural or functional integrity of ATG7 as an E1-enzyme. In summary, the data indicates that a novel region emerged early in vertebrates in the highly conserved AD of ATG7. Comparison with other members of the E1-enzyme family showed that ATG7 is unique in this respect, as the AD has remained conserved in other members of this protein family. Despite gaining a new region, the rest of the AD has remained highly conserved.

### The VSR has experienced purifying selection, with the exception of one cancer-related site that was shaped by positive selection in mammals

**Figure 3.**
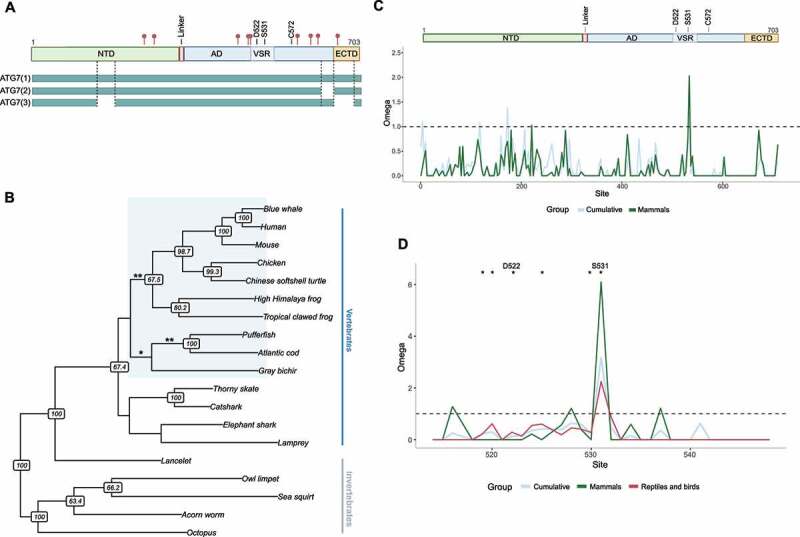
(**A**) Schematic overview of ATG7, including locations of SNPs related to diseases in humans (red pins). The protein mainly consists of two globular domains: a N-terminal domain (NTD) and a C-terminal domain that is further divided into the adenylation domain (AD) and an “extreme C-terminal ATG7-specific” domain (ECTD). ATG7 has a single catalytic cysteine residue, C572, instead of a separate catalytic cysteine domain that characterizes the canonical E1-enzymes [12,14,15]. Three protein-coding isoforms of ATG7 have been identified, ATG7(1-3). The vertebrate-specific region (VSR) is situated within the AD and harbours two cancer-related sites. A missense variant, D522E, has been linked to increased risk of hepatic cancer in humans [17]. S531 has been found to be phosphorylated in human cancers [39]. Other disease-related variants are indicated with red points [18,19] (**B**) Analysis of positive selection on specific branches of ATG7, in a phylogeny of vertebrate taxa and some of their most related invertebrate relatives (using the aBSREL model, see methods). ATG7 went through three notable phases of accelerated evolution: in the ancestor of bony fish (p = 0.039), the ancestor of teleosts (p = 0.0018), and the ancestor of tetrapods (p = 0.0132). (**C**) Sliding window analysis of selection in ATG7. The x-axis shows amino acid site in ATG7 and the y-axis shows omega values or the dN/dS ratio. Step size 9 bp. Green line includes exclusively mammalian branches of the phylogeny and the blue line includes all branches. The schematic of ATG7 above shows where each domain is situated (NTD, AD and VSR, and ECTD), the catalytic cysteine (C572) and the two cancer-related sites (D522 and S531). (**D**) Sliding window analysis showing the region between L512-K548, amino-by-amino acid. *p < 0.1 (only including sites that are significant in mammals but not other tetrapods). In addition to (**C**), reptiles and birds were grouped and are shown in red.

We were interested in the putative biological functions of the VSR region in ATG7. Alignment shows where the VSR resides within the AD and crucially that it is present in all three protein coding isoforms of human ATG7 (Figure 3A). Interestingly, one disease-related variant resides within the VSR. This variant, D522E, has been linked to hepatic cancer in humans [17]. Moreover, ATG7 has number of sites found to be post-translationally modified in human diseases. One such site, S531, resides in the VSR. Phosphorylation of S531 was detected in tumors in a proteogenomics study of invasive breast adenoma [39].

Novel protein domains are expected to evolve more rapidly than ancient and highly conserved domains, because the molecular structures may require further adaptive tweaks to achieve optimal biological function [41]. Thus, if the VSR emerged early among vertebrates, one might expect positive selection to shape the diversity in ATG7 on specific branches. We analyzed ATG7 sequences on a phylogeny, ranging from tunicates and cephalochordates to mammals, using the branch-site model aBSREL [40]. This model can implicate specific branches of the phylogeny that may have experienced diversifying positive selection. The data revealed that ATG7 went through three notable phases of accelerated evolution, in the ancestor of tetrapods, that of fishes and on the branch leading to bony fish (Figure 3B and table S4 for details from the aBSREL analysis). Inspection of the alignments of bony- and cartilaginous fishes indicates evolution of an even longer region than in the other vertebrates (figure S3A, the expansion was in bony fishes and the most related cartilaginous fish, elephant shark). Further analyses of more fish species and mammals showed that most species of bony fish have evolved a similar serine-rich region, and this emergence seems to have begun in cartilaginous fish (figure S3B). Interestingly, the exception was gray bichir (*Polypterus senegalus*), a bony fish whose ancestors separated from teleosts before the teleost-specific whole-genome duplication [42].

Because the VSR emerged in vertebrates and the rapid evolution of ATG7 close to the base of that group, it was of interest to study the evolution of ATG7 and this domain further. Sliding window analysis of dN/dS ratios on a site-by-site basis was done using the FEL model (Figure 3C-D), in 21 tetrapod lineages. Sites with p < 0.1 were considered significant under this model [43]. This can implicate specific residues that may have experienced positive selection and enables site-specific comparison of evolutionary dynamics between mammals and other vertebrates. Predictably, most sites were under purifying selection. However, one site (S531 in the human gene), was under significant positive selection in mammals. Interestingly, this was the site where post-translational modifications (PTM) have been detected in cancers as discussed above.

No site was under significant positive selection in other tetrapods. Moreover, the cancer-related site (D522, [17]), was found to be under significant purifying selection in mammals, but not in other tetrapods. For comparison, the catalytic cysteine residue, C572, is under significant negative selection in all groups, as expected. A few sites, close- or adjacent to D522 and S531 were also found to be under significant negative selection in mammals only. This suggests the function of this key amino acid (D522), and seemingly a sizeable fraction of the VSR, had become a polished adaption that purifying selection maintains in mammals. In summary, ATG7 went through three notable phases of accelerated evolution in the ancestor of tetrapods and fish. The VSR seems to be maintained by purifying selection in vertebrates, suggesting a functional role of the region, apart from one site which is under significant positive selection in mammals. That site, S531, and another site in the region, D522, have been linked to cancer in humans.

### The new vertebrate specific region is predicted to be intrinsically disordered

**Figure 4.**
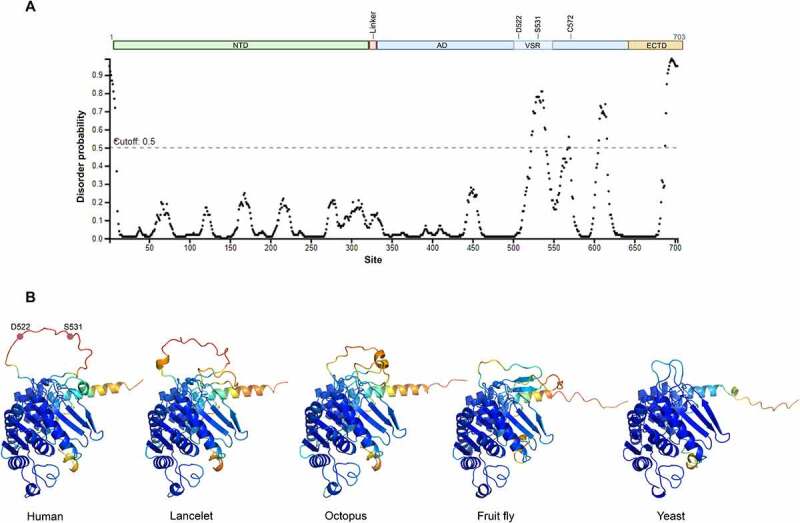
(**A**) Prediction of disorder in human ATG7. The y-axis shows disorder probability, where values above 0.5 (dotted line) are considered disordered. The x-axis shows site in the protein. The schematic above depicts each domain of the protein (NTD, AD and the VSR, and ECTD). The prediction was made with Pspred [46]. (**B**) Structural models of ATG7, comparing humans, lancelet, octopus, fruit fly, and yeast. The comparison shows how the VSR emerged gradually as an intrinsically disordered loop. The figures focus on the C-terminal domain and show the protein as a monomer for clarity. The structures are based on AlphaFold predictions. The two cancer-related sites D522 and S531 are indicated on the human structure. The residues are color-coded as in the AlphaFold structure database, according to their respective per-residue confidence score (pLDDT), where blue color is the highest confidence and orange is the lowest.

To study the potential structure and function of the vertebrate-specific region we used structure models obtained with AlphaFold [44] and disorder predictions from other programs (Figure 4A). The models predict the VSR forms an intrinsically disordered loop that turns outward in the opposite direction of the catalytic cysteine residue, leaving it open for interactions with other proteins or molecules. Several other tools that predict protein disorder also suggest the VSR is most likely intrinsically disordered [45–50] (Figure S4). Moreover, the flDPnn server [49] predicts that the VSR contains a possible macro molecular binding site (Figure S4C).

To visualize the structural changes occurring as the VSR emerged in evolution, we compared structural models from a few distantly related species, from yeast to humans (Figure 4B). The comparison shows how the VSR seems to have emerged by elongating steadily through eukaryotic evolution rather than in a single event. As mentioned before, fish have evolved a longer serine-rich VSR. This fish-specific development is clearly seen in the zebrafish protein structure as an even longer intrinsically disordered loop (figure S4C).

### The VSR is important for the lipidation of LC3

**Figure 5.**
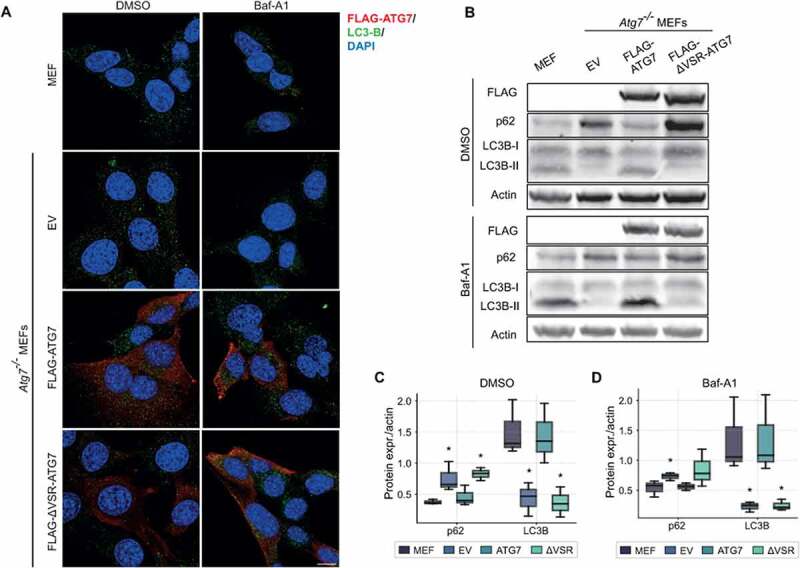
**(A)** WT MEFs and *Atg7^−/−^* MEFs expressing FLAG-tagged full length ATG7 and ATG7 lacking the VSR (ΔVSR-ATG7), or empty vector (EV) were treated either with Bafilomycin-A1 (Baf-A1) or DMSO for 4 h. The cells were stained for DAPI (blue), LC3B (green) and FLAG (red). The scale bar shows 10 µm. **(B)** Western blot analyses from the same set of cell lines and treatments as in (A). The cells were stained for FLAG, LC3B, p62, and actin. **(C-D)** Quantification of band intensities in cells treated with DMSO (C) and Baf-A1 (D). The band intensities of p62 and LC3B were normalized against actin. The error bars represent SEM of three independent experiments. Statistical significance of *Atg7^−/−^* MEFs expressing ATG7, ΔVSR, or EV, compared to WT MEFs was determined by Mann-Whitney U-test. *p < 0.05.

To address whether the VSR affects autophagy, we used *Atg7* knockout mouse embryonic fibroblasts (MEFs) and generated stable cell lines with doxycycline inducible expression of either full length ATG7 or with a deletion of the VSR (ΔVSR-ATG7). Confocal imaging showed a similar, mainly cytoplasmic, expression pattern of WT ATG7 and ΔVSR-ATG7 (Figure 5A). No clear differences in LC3B puncta were observed between the different cell lines, even when treated with the autophagy degradation inhibitor Bafilomycin-A1 (Baf-A1). This was, however, not surprising as we have observed similar LC3 puncta patterns before, despite clear effects on LC3 lipidation [16]. As expected, immunoblotting revealed that *Atg7^−/−^* cells are unable to lipidate LC3 (Figure 5 B-D). This phenotype was rescued by introducing expression of WT ATG7. Interestingly, ATG7 lacking the VSR fails to rescue the lipidation of LC3. Moreover, these cells exhibited accumulation of p62. These results indicate an important role of this region in autophagy and for the function of the AD.

## Discussion

Autophagy was a major innovation in evolution, that gave eukaryotes unique capabilities for maintaining cellular homeostasis by recycling intracellular components. The process helps cells survive under stress, such as nutrient- or oxygen deprivation, and prevents the accumulation of defective cellular material [1]. This important role of autophagy is reflected in its relation to cancer, where autophagy has tumor suppressing effects by maintaining cellular homeostasis – but after a tumor has formed, cancer cells utilize autophagy to survive within the challenging tumor environment [38]. About 20 genes encode the core machinery for autophagy, but there is substantial variation in their numbers and composition between taxa. In this study, we conducted evolutionary comparisons of ATG7 from unicellular eukaryotes to mice, which indicates that a novel region emerged within ATG7 early in vertebrates. The emergence of the domain seems to have been gradual, thus there was not a single event of origin. Analyses of selection show that this vertebrate-specific region (VSR) is mostly under strong purifying selection in tetrapods. Additional analyses in fish showed that this region is highly conserved between bony fishes. This indicates that the VSR stabilized early in vertebrates and suggests a functional role.

Interestingly, this region is situated within the adenylation domain (AD) of the protein. This is the active domain of the protein and is highly conserved in all E1 enzymes. The AD harbors one of the most highly conserved function of ubiquitin enzymes, the formation of a covalent bond to a protein-substrate [9–11]. Comparison with other E1-enzymes showed that ATG7 is unique in this respect as the AD of other members of this protein family have been under strong evolutionary constraints from yeast to humans. Despite gaining a new region the AD of ATG7 has remained highly conserved in other parts of this domain with 71% percentage similarity between yeast and humans. This implies that the addition of this new domain has not interfered with the structural or functional integrity of ATG7 as an E1-enzyme. Interestingly, the vertebrate specific region (VSR) contains a site where we previously discovered a missense variant, D522E, that confers an increased risk of hepatocellular- and cholangiocarcinoma in humans [17]. That further confirms the functional importance of the domain.

Novel protein domains are shaped by positive selection and are expected to evolve more rapidly than ancient and highly conserved domains. To explore when in the evolutionary history of vertebrates from their ancestors the VSR emerged we analyzed the gene in species ranging from cephalochordates and tunicates to mammals using the aBSREL model. This model indicates diversifying positive selection, *i.e*. accelerated evolution, in specific branches of the phylogeny. The analysis revealed that entire ATG7 went through three episodes of accelerated evolution, in the ancestor of tetrapods, bony fish, and again in teleosts. Furthermore, the aBSREL analysis also showed that bony fish and a close cartilaginous relative of bony fish have evolved an even longer VSR than other vertebrates. Further investigation indicated that teleosts have evolved a longer region, in the exact same location in ATG7 (S7A-B). This emergence seems to have begun in cartilaginous fish and become a conserved serine-rich region within the bony-fish clade. Though the VSR seems to have emerged gradually throughout eukaryotic evolution, this data suggests two separate evolutionary phases in tetrapods and teleosts. Further exploration of the function of ATG7 in fishes could shed light on the functional importance of this domain in that group. The extra genomic duplications at the base of fishes and within the salmonid ancestor may provide opportunity for exploring the functional evolution of VSR domains [52,53].

Site-by-site analysis of natural selection revealed that the protein and the VSR are mostly under purifying selection, confirming the functional importance of the domain, probably since its emergence in the earliest vertebrates. The cancer-related site, D522, was under significant negative selection specifically in the mammalian clade. A study on mice with tissue-specific knockout of *Atg7* in hepatocytes revealed that they develop hepatocellular carcinoma, similar to what we have found to be correlated with the D522E variant [17,51]. However, the analysis also revealed strong positive selection on one site within the VSR. This site, S531, was under significant positive selection only in the mammalian clade, though some indication of a signal was found in other tetrapods. This can also be seen in the alignment from the same group of species, where a serine is present at this site in most mammalian species while there is more variation in other tetrapod species (figure S3D). This residue is not conserved in fish (figure S3A-B). Interestingly, this site has a reported disease link as it was found phosphorylated in tumor samples in a study of invasive breast adenoma [39].

To explore the structure of the VSR we used the protein structure database Alpha fold. Interestingly, the region is predicted to be intrinsically disordered. This was further supported by other predictive tools [45–50]. Structural comparison between distantly related eukaryotic species showed that the region emerged gradually from yeast to humans and forms an even longer intrinsically disordered loop in zebrafish (figure S3C). Moreover, the VSR was predicted to contain a macro-molecular binding site. The structural models show that the VSR loop turns outwards from the protein, leaving it open to interactions with other molecules. Protein domains are generally structurally conserved as even a change in a single amino acid can affect the folding process. Therefore, it is hypothesized that protein domains evolve through gradual changes at the periphery of the protein core, while leaving the domain core structures mostly unchanged [41]. The structural models and the alignments suggest that the VSR has not affected the structural integrity of ATG7, but rather emerged as an extension from the AD.

Studies have shown that lack of Atg5 or Atg7 is neonatal lethal in mice, while fruit flies are viable [30, 54, 55, 61 62]. For instance, *Atg7* deficient mice were born histologically normal, apart from reduced birthweight and lower amino acid levels [55]. However, they died within one day of birth. Though the reason is unclear, the authors speculate whether ubiquitin-positive inclusions found in some organs of the mutant mice at birth could be involved. In contrast, *Atg7* is dispensable during development in *D. melanogaster* [30]. The study showed that Atg7 deficient flies were viable despite severe defects in autophagy. The development of these flies proceeded normally, although slower during the pupal stage. The adult flies had reduced lifespan and were hypersensitive to both nutrient and oxidative stress. Moreover, similar to mice, accumulation of ubiquitin-positive aggregates was found in degenerating neurons. Zebrafish with *Atg7* morpholino knockdown show severe developmental abnormalities, both in body morphology and heart development, and reduced lifespan. This indicates that ATG7 is important during development of other vertebrate species [56]. These examples suggest a more important function of autophagy during development in mice and perhaps mammals or vertebrates.

Several autophagy-independent functions of ATG7 have been described (discussed in more detail below) [20, 22]. These have exclusively been described in mammalian models. Therefore, it is unknown whether other taxa share these functions. Moreover, novel functions of ATG7 have not been assigned to any specific region of the protein. We wondered whether the VSR could harbor an autophagy independent function.

ATG7 conveys its dual role in autophagy through its E1-like enzymatic properties. The shorter isoform of ATG7 has been shown to bind the tumor suppressor p53 [24]. This isoform, ATG7(2), lacks amino acids necessary for lipidation of LC3/GABARAP. The binding of ATG7(2) to p53 was not lost when the catalytic cysteine (C572) was substituted and a 40 amino acid region on the C-terminal, which has been shown to be necessary for the E1-enzymatic function of ATG7 [57], was deleted. This binding was therefore independent of the E1-like enzymatic activity of ATG7. By binding p53, ATG7 regulates the transcription of cell cycle inhibitor p21^CDKN1A^. Lack of ATG7 resulted in failure to undergo cell cycle arrest and increased p53-dependent apoptosis [24]. ATG7 has been implicated in cell migration and adhesion in several studies. Work on chick embryos revealed that ATG7 promotes E-cadherin expression delaying EMT. This was further confirmed in human colorectal carcinoma cells (HCT116) [25]. Interestingly, that study was based on the same ATG7(2) construct as was used in the study on p53 discussed above. In agreement with this study, ATG7 was shown to inhibit EMT in triple-negative breast cancer cells (TNBC) by upregulation of E-cadherin and downregulation of N-cadherin, SMA, and Vimentin. This in turn led to decreased proliferation and migration of TNBC and increased apoptosis [26].

A few other autophagy-independent roles of ATG7 that have been described rely on ATG5-ATG12 conjugation and, thus, its E1-like enzymatic properties. These include functions related to cell secretion and innate immunity, where ATG7 has been shown to participate in bone degradation, by mediating the release of lysosome-residing resorptive molecules by osteoclasts, and LC3-associated phagocytosis (LAP), where a few autophagy proteins participate in the degradation of a wide range of intracellular pathogens without forming an autophagosome. Instead, the phagosome fuses directly with lysosomes [32,34]. Moreover, ATG7 has been shown to be required for IFN-γ dependent blockage of membrane rearrangements, which occurs during replication of certain pathogens [33]. A few other autophagy independent roles of ATG7 have been described, but not explored whether they are dependent on its E1-like enzymatic activity or not. These include, forming a complex with caspase-9, repressing its proteolytic activity and thereby preventing apoptosis, and inducing phosphorylation of MLKL leading to necrosis [27,28]. Further studies in more mammalian and vertebrate model organisms will be important for shedding light on the evolutionary history and relationship of these novel ATG7 functions.

The main function of ATG7 in autophagy is to facilitate the lipidation of LC3/GABARAPs. To address whether the VSR affects this function of ATG7 we used *Atg7^−/−^* MEFs rescued with doxycycline inducible expression of either full length ATG7 or lacking the VSR (ΔVSR-ATG7). Interestingly, the results revealed that while the full length ATG7 is able to rescue the lipidation of LC3B, ΔVSR-ATG7 is not. These results indicate an important role of this region in autophagy and for the function of the AD. Further characterization of this cell model will help reveal the relationship between this region and autophagy in vertebrates.

## Materials and methods

### Alignments

All coding DNA sequences and amino acid sequences used were obtained through the Ensembl database (www.ensembl.org), from NCBI (www.ncbi.nlm.nih.gov), and through Orthodb (www.orthodb.org). Multiple sequence alignments, both for DNA- and amino acid sequences, were made using Clustal Omega [61] and adjusted manually using JalView (version 2.11.1.6), to maintain the correct reading frame. Phylogenetic trees were constructed in R (version 4.1.1) using the Ape (version 5.6-1) and Phangorn (version 2.8.1) packages [59,60]. The optimal tree was obtained using maximum likelihood and neighbor joining with 1000 bootstrap replicates. The trees were constructed using the JTT model, where a matrix of pairwise distances is created.

For comparison of E1 family enzymes, the adenylation domain from all E1- and E1-like enzymes possessing an active adenylation domain were included. The transcripts are listed in table S3. For analyses of ATG7, nine distantly related species ranging from yeast to humans were initially aligned. The sequence IDs are listed in table S1.

### Tests for signatures of natural selection

Several approaches were used to test for direction and intensity of natural selection within the gene across the phylogeny. The aBSREL (adaptive branch-site random effects likelihood) model (www.datamonkey.org/absrel, run date April 2021 [50]) was used to evaluate whether positive selection had occurred on individual branches of the tested phylogeny. The model is based on the average omega values (dN/dS) for the tested protein, on each branch [56]. This was run on the entire ATG7 gene, for 19 species dataset including tunicates and cephalochordates, such as sea squirt and lancelet, to mammals. These species were chosen to evaluate whether accelerated evolution of ATG7 occurred at the border of invertebrates and vertebrates, therefore including the closest vertebrate relatives. Moreover, additional species of fish were aligned for comparison of the VSR between fish and tetrapods. The DNA sequences and transcripts are listed in table S5. The species were chosen to represent a balanced group between vertebrates and close vertebrate relatives, the challenge being lack of reliable sequencing data from invertebrate species.

The ratio of replacement to silent polymorphism (dN/dS ratios, omega) was calculated for a vertebrate test group, which included 21 tetrapod species (DNA sequences are listed in table S8), by the FEL (fixed effects likelihood) model [43] and through the Datamonkey online bioinformatics tool (datamonkey.org/fel, run date August 2020). The model is phylogeny based, and selective pressure is assumed to be constant along the phylogeny. The dN/dS ratios are determined directly for each site using maximum likelihood approach. The raw data from the FEL analysis was then used to construct a sliding window in R. Sites with undetermined dN/dS values or a dN/dS of infinity (meaning that dN equals zero at that site) were excluded, as a definite value is necessary for generating a plot. Excluding these sites did not affect significance calculations as those were based on the raw data and made automatically by the Datamonkey server.

### Structural predictions

Structural models were obtained from the Alpha fold protein structure database (alphafold.ebi.ac.uk) when the models of the corresponding species were available or using the ColabFold platform (https://github.com/sokrypton/ColabFold) entering the respective sequences. Structure figures were generated with the software PyMOL (http://pymol.org). The prediction of intrinsic disorder was further confirmed by other predictive tools. Those were IUPred [45] (https://iupred2a.elte.hu), Pspred [46] (http://bioinf.cs.ucl.ac.uk), PrDOS [47] (https://prdos.hgc.jp), ESpritz [48] (http://old.protein.bio.unipd.it), flDPnn [49] (http://biomine.cs.vcu.edu/servers/flDPnn/), and Odinpred [50] (https://st-protein.chem.au.dk/odinpred). In addition, flDPnn was used to predict putative macro-molecular binding sites and PhosphoSitePlus [58] (https://www.phosphosite.org/homeAction) was used to assess whether any PTM could be detected within ATG7 (results not shown). The predictions are shown in supplemental figure S4.

### Cell lines and analyses

*Atg7^−/−^* MEFs were a gift from Masaaki Komatsu and cultured as described previously [55]. The cells were seeded onto 12-well plates at a density of 500 × 10^5^ cell per well for Western blot analyses or at a density of 9 x 10^3^ cells per well for confocal imaging. The WT ATG7 construct was generated as described in [16] and then the VSR was deleted from that construct to generate ΔVSR-ATG7. The region was deleted using the Q5 Site-directed Mutagenesis Kit (New England Biolabs) with the following primers: ΔVSR F, 5’-CTTGGCTGCTACTTCTGC-3 and ΔVSR R, 5’-ACCATGTCTCATGACAACAAATG-3’. The constructs were verified by Sanger sequencing. The cells were incubated overnight in doxycycline. Cells expressing full length ATG7 received 0.1 µg/mL of doxycycline while cells expressing ΔVSR-ATG7 or EV received 0.5 µg/mL. Then the media was replaced with either 100 nM Bafilomycin-A1 or vehicle control (DMSO) and incubated for 4 h.

Western blot analyses were carried out as described previously [16]. The primary antibodies used were mouse anti-FLAG (Sigma, F3165, 1:5000), mouse anti-actin (Millipore, MAB1501, 1:15000), rabbit anti-LC3B (Cell Signaling, #2775, 1:1000), and rabbit anti-p62 (Cell Signaling, #5114, 1:1000). The band intensities were quantified in Image J and statistical analyses were conducted using Python. Statistical significance was determined by Mann-Whitney U-test. P values < 0.05 were considered significant. Three independent experiments were carried out.

For confocal imaging, the cells were fixed with 4% paraformaldehyde for 15 minutes, washed with PBS for 3x 5 minutes, and incubated in blocking buffer for 1 h at room temperature (RT). The washing step was repeated before the cells were incubated overnight with the primary antibodies. The primary antibodies used were mouse anti-FLAG (Sigma, F3165, 1:5000), rabbit anti-LC3B (Cell Signaling, #2775, 1:5000). The cells were washed again before the secondary antibodies were applied and incubated for 1-2 h. The slides were mounted with Fluoromount-G^TM^ with DAPI (Invitrogen). The slides were imaged on a confocal microscope (Olympus FLV1200). Three independent experiments were carried out.

## Supplementary Material

Supplemental Material
