# Supplementary material for: A novel region within a conserved domain in ATG7 emerged in vertebrates
Source: Autophagy Rep. 2022 Sep 7;1(1):393–413. doi: 10.1080/27694127.2022.2118933 (PMC11864663; doi:10.1080/27694127.2022.2118933)
Supplement: Supplemental Material [file KAUO_A_2118933_SM3441.zip › FigureS1.pdf]

```
Yeast/1-294         ---MNDIKQLLWNGELNVLVSIDPSFLMKGSPREIAVLRIRVPRETYLVNYMPLIWNKIK
Roundworm/1-275     --MDYEVCRKVWESHVPCQFTLQSSGGTHG--EP-LPFYTMLPR----FSYLALAIQKVL
Fruit fly/1-269     MAHDREVLRMIWEGQIGICFQADRDEIVGI--KP-EPFYLMISR----LSYLPLVTDKVR
Octopus/1-268       MAEDREILREVWEGRIPVSFSLSPDEIVNEQPDP-FFYYLLVPR----LSYFPLVTDKVQ
Lancelet/1-273      MADDREVLRELWDGRLPIKFNLAADEVSSM-EHP-EPCYLLVPR----QSYFPLVADKVQ
Coelacanth/1-275    MSDDKDVLRDVWFGRIPTCFTLYQDEMTER--EA-EPYYLLLPR----VSYLTLVTDKVK
Frog/1-233          MTDDKDVLRDVWFGRIPTCFTLYQDELTER--EA-EPYYLLLPR----ISYLTLVTDKVK
Mouse/1-275         MTDDKDVLRDVWFGRIPTCFTLYQDEITER--EA-EPYYLLLPR----VSYLTLVTDKVK
Human/1-275         MTDDKDVLRDVWFGRIPTCFTLYQDEITER--EA-EPYYLLLPR----VSYLTLVTDKVK

Yeast/1-294         SFLSFDPLTD--SEKYFWFEHNKTPIPWNYPVGVLFDCLAGKSATFTTSFENQVKDVLTF
Roundworm/1-275     SSFNRRDDGEKVHSDKMWLEHNGIPLKMYIPIGVIYDQANLSENDSILEIIVRT------
Fruit fly/1-269     KYFSRYISAEH-QDGAVWFDFNGTPLRLHYPIGVLYDLLHPEEDSTPWCLTIHF------
Octopus/1-268       KYFQKFLDAE--IQGEFWLEFEGQPLKWHYPVGVLFDLYG-SEENLPWKLTVHF------
Lancelet/1-273      RYFLKYTANS--QGEEMWLEYEGQPLKWHYPIGVLFDLFA-SSTMLPWSLVVHF------
Coelacanth/1-275    KHFQKVMRPE--DIGEAWFEYEGTPLKWHYPIGLLFDLHA-SNTALPWSIVVHF------
Frog/1-233          KHFQKVMRTE--DISEIWFEFEGIPLKWHYPIGLLFDLHA-SNTSLPWSITVHF------
Mouse/1-275         KHFQKVMRQE--DVSEIWFEYEGTPLKWHYPIGLLFDLLA-SSSALPWNITVHF------
Human/1-275         KHFQKVMRQE--DISEIWFEYEGTPLKWHYPIGLLFDLLA-SSSALPWNITVHF------

Yeast/1-294         LRIHLVMGDSLPPTIIPIASSKTQAEKFWFHQWKQVCFILNGSSKAIMSLSVNEARKFWG
Roundworm/1-275     --------SQPPP--QFQMVDRDMMEAMFMQNIKE-ADYLKTKAEITKNMMKDESAQLWR
Fruit fly/1-269     --------SKFPEDMLVKLNSKELLESHYMSCLKE-ADVLKHRGLVISAMQKKDHNQLWL
Octopus/1-268       --------QNFPKDELLHCPNKDAVEAHFMSMVKE-ADALKHRSQVINGMQKKDHKQLWL
Lancelet/1-273      --------QKFPEDELLHCPGKDAVESHFMSSVKE-ADTLKHRGQVINGMQKKDHKQLWM
Coelacanth/1-275    --------KNFPDKDLLHCPSKDVIEAHFMSCIKE-ADALKHKSQVINEMQKKDHKQLWM
Frog/1-233          --------KNFPAKDLLHCQSKDVIEAHFMSSVKE-ADALKHKSQVINEMQKKDHKQLWM
Mouse/1-275         --------KSFPEKDLLHCPSKDAVEAHFMSCMKE-ADALKHKSQVINEMQKKDHKQLWM
Human/1-275         --------KSFPEKDLLHCPSKDAIEAHFMSCMKE-ADALKHKSQVINEMQKKDHKQLWM

Yeast/1-294         SVIT--RNFQDFIEISNKISSS-----RPRHIPLIIQTSRTSGTFRISQ-PTISMTGVNP
Roundworm/1-275     SVCNIPDNFDEFWTIVQKLMET-SEGNEFAHIPLRVYVK--NQAFKQALITAKHPDGSLR
Fruit fly/1-269     GLVN--EKFDQFWAVNRRLMEPYGDLESFKNIPLRIYTD-DDFTYTQKLISPISVGGQKK
Octopus/1-268       GLLN--DKFDQFWSVNRKLMES-GEE-KFKYIPYKIYLS--DGSFSQQLFKPIDENGTVH
Lancelet/1-273      GLQN--DKFDQFWAINRRLMEH-GEENCFKHLPFRLYQP--DKPCVQRLFRPITDEGEQR
Coelacanth/1-275    GLQN--DKFDQFWAINRKLMECPADENGFRYIPYRIYQATSERPFIQKLFRPFTADGQPH
Frog/1-233          GLQN--DKFEQFWAINRKLMEVPPEDGGFRYIPFRIYQAINERPFIQKLFRPVANDGRPY
Mouse/1-275         GLQN--DRFDQFWAINRKLMEYPPEENGFRYIPFRIYQTTTERPFIQKLFRPVAADGQLH
Human/1-275         GLQN--DRFDQFWAINRKLMEYPAEENGFRYIPFRIYQTTTERPFIQKLFRPVAADGQLH

Yeast/1-294         TLKDIEGDILDVKEGI-----NGNDVMVICQGIEIPWHMLLYDLYSKLRSFDGFLYITLV
Roundworm/1-275     TIGEAVSDVLSSSSSSSTDSQSEHPPRLISHGIDIPHHTPLIFAAKNLSYPDNFIHVVLL
Fruit fly/1-269     SLADLMAELSTPVRRA---------VGCRTHGIDLHEETQLQWMSEHLSYPDNFLHLSVD
Octopus/1-268       LLSDLMQQALPDFNGDE----TKLKPQVFIQGIEVPWETPILWLCEHLSYPDNFLHICVH
Lancelet/1-273      LLGDLVREVAPQVFNTEA-EDASGRWKVVIQGVEPPMETPVQWLSEHFSYPDNFLHICLV
Coelacanth/1-275    TLGDLLREVFPAAITPE---DSEKKFQVVIHGIEPMLETPVQWLSEHLSYPDNFLHISII
Frog/1-233          TLGDLIREVCPAAVPAE---GHV-------------------------------------
Mouse/1-275         TLGDLLREVCPSAVAPE---DGEKRSQVMIHGIEPMLETPLQWLSEHLSYPDNFLHISIV
Human/1-275         TLGDLLKEVCPSAIDPE---DGEKKNQVMIHGIEPMLETPLQWLSEHLSYPDNFLHISII

Yeast/1-294         PIKGGDKASSEL
Roundworm/1-275     LVVP--------
Fruit fly/1-269     YKDV--------
Octopus/1-268       Q-----------
Lancelet/1-273      NSQ---------
Coelacanth/1-275    PQPTD-------
Frog/1-233          ------------
Mouse/1-275         PQPTD-------
Human/1-275         PQPTD-------
```
