# Supplementary material for: A novel region within a conserved domain in ATG7 emerged in vertebrates
Source: Autophagy Rep. 2022 Sep 7;1(1):393–413. doi: 10.1080/27694127.2022.2118933 (PMC11864663; doi:10.1080/27694127.2022.2118933)
Supplement: Supplemental Material [file KAUO_A_2118933_SM3441.zip › FigureS2.pdf]

```
S cerevisiae/1-206      ------------------------------------------------------------
S pombe/1-268           ---------------------------MPSSDVCKAGS----HRHSGWIQSLKKPGPFNL
Owl limpet/1-255        ------------------------------------MAVNWPRRWGHVQKLTERSGPFAH
Sea squirt/1-262        ---------------------------MEVD-DC-DDGARWSGRWNHLQKILERNGPFVH
Roundworm/1-257         ---------------------------------MVSVDPLATERWRSIRRLTDRDSAYKV
Fruit fly/1-262         ---------------------------MSVHSPN-PGLILQSKRFNGLRNILEREGPFCK
Octopus/1-269           ----------------------MLSKQTTTNWSVKRMESEWGDRWSHLKKILERSGPFTH
Lancelet/1-273          -------------------MANQAEEPMMVDGRVDSPGGSWPDRWNHVKKLLERAGPFAH
Lamprey/1-281           ---------MAAEPEKKRSRVEEHPHNMAVESGC-VESMDWDGRWNHISKFLERPGPFTH
Gray bichir/1-290       MRWAPENMAEALEPEKKRRRIEELTDKMAVDGGC-GDTGDWEGRWNHVKKFLERSGPFTH
Elephant shark/1-283    -------MADVEAPEKKRRKVEEHSDKMSVDGGY-GDTGDWEGRWSHVKTFLERSGPFTH
Zebrafish/1-283         -------MAEGEEPEKKRRRIEELNEKMVVDGGS-GDRSEWQGRWDHVRKFLERTGPFTH
Frog/1-263              ---------------------------MAVDGGC-GDTGDWEGRWNHVKKFLERSGPFTH
Mouse/1-263             ---------------------------MAVDGGC-GDTGDWEGRWNHVKKFLERSGPFTH
Human/1-263             ---------------------------MAVDGGC-GDTGDWEGRWNHVKKFLERSGPFTH

S cerevisiae/1-206      --------------MDCKILVLGAGGLGCEILKNLTMLSFVKQVHIVDIDTIELTNLNRQ
S pombe/1-268           DAPENPEETLKSAF-SSKILIIGAGGLGCEILKDLALSGF-RDLSVIDMDTIDITNLNRQ
Owl limpet/1-255        PDFEASEETLTFLQDTCHILIIGAGGLGCELLKDLVLLGI-RNIDVIDMDTIDVSNLNRQ
Sea squirt/1-262        PDFEPSPDILDFMLNSCKVLVVGAGGLGCELLKDLSLMGL-RHIHVIDMDTIDLSNLNRQ
Roundworm/1-257         PWFVPGPENFEALQN-TKILVIGAGGLGCELLKNLALSGF-RTIEVIDMDTIDVSNLNRQ
Fruit fly/1-262         DGFAASSENLEFLQTKCQVLIIGAGGLGCELLKDLALMGF-GNLHVIDMDTIELSNLNRQ
Octopus/1-269           SDFEPSPETLIFLHETFRILIVGAGGLGCELLKNLALLGV-GNIDIIDMDIIDISNLNRQ
Lancelet/1-273          PDFEPSPETIQFMLEVCKILCIGAGGLGCELLKDLALMGF-RNIHVIDMDKIDVSNLNRQ
Lamprey/1-281           PDFEPSTETLSFLLESCKILVLGAGGLGCELLKDLALSGF-RQIHVIDMDTIDVSNLNRQ
Gray bichir/1-290       PDFEPSTESLGFLLNTCKILVLGAGGLGCELLKNLALSGF-RQIDVIDMDTIDVSNLNRQ
Elephant shark/1-283    PDFEPSTELLQFLLETCKVLIIGAGGLGCELLKDLALTGF-RQIDIIDMDTIDVSNLNRQ
Zebrafish/1-283         PDFEASTESLQFLLDTCKILVIGAGGLGCELLKDLALSGF-RHIHVVDMDTIDVSNLNRQ
Frog/1-263              PEFEPSNESLQFLLETCKILVVGAGGLGCELLKNLALSGF-RQIHVIDMDTIDVSNLNRQ
Mouse/1-263             PDFEPSTESLQFLLDTCKVLVIGAGGLGCELLKNLALSGF-RQIHVIDMDTIDVSNLNRQ
Human/1-263             PDFEPSTESLQFLLDTCKVLVIGAGGLGCELLKNLALSGF-RQIHVIDMDTIDVSNLNRQ

S cerevisiae/1-206      FLFCDKDIGKPKAQVAAQYVNTRFPQLEVVAHVQDLTTLPPSFYKDFQFIISGLDAIEPR
S pombe/1-268           FLFNESNIDEPKANVAASMIMKRIPSTVVTPFYGKIQDKTIEFYKEFKLIICGLDSVEAR
Owl limpet/1-255        FLFRPKDVGKTKAETAANFINQRIPGANVKAYFAKIQDYDTDFYRKYHIIVCGLDSIVAR
Sea squirt/1-262        FLFRQCDIGKPKAEVAASFINKRVKGTKCVPHFCRIQDHDSSFYKQFHIVVCGLDSIVAR
Roundworm/1-257         FLFRESDVGKSKAEVAAAFVQQRVVGCQVTAHNCRIEDKGQEFYRKFSIIICGLDSIPAR
Fruit fly/1-262         FLFRRTDIGASKAECAARFINARVPTCRVTPHFKKIQDFDESFYQQFHLVVCGLDSIVAR
Octopus/1-269           FLFRPKDVGKSKAEVAAEFINTRITNCKVKPHFAKIQDFPDSFYQQFHVVVCGLDSIVAR
Lancelet/1-273          FLFRPKDVGKPKAETAAEFVNTRVPGCRVTPHYAKIQDFDGEFYRQFHIIVCGLDSILAR
Lamprey/1-281           FLFRPKDVGRPKANVAAEFINTRVPGCNVMPHFKKIQEYDESFYRQFHIIVCGLDSIVAR
Gray bichir/1-290       FLFRPKDVGRPKAEVAAEFINSRIPGCNVTPHFKKIQDFDETFYKQFHIVVCGLDSIIAR
Elephant shark/1-283    FLFRSKDVGRPKAEVAADFINSRIPGCNVVPHFKKIQDFSDTFYRQFHIVVCGLDSIIAR
Zebrafish/1-283         FLFRPKDVGRPKAEVAADFVNDRVPGCSVVPHFKKIQDLDETFYRQFHIVVCGLDSVIAR
Frog/1-263              FLFRPKDVGRPKAEVAAEFINTRIPDCCVTPHFTKIQDFDETFYREFHIIVCGLDSIIAR
Mouse/1-263             FLFRPKDVGRPKAEVAAEFLNDRVPNCNVVPHFNKIQDFNDTFYRQFHIIVCGLDSIIAR
Human/1-263             FLFRPKDIGRPKAEVAAEFLNDRVPNCNVVPHFNKIQDFNDTFYRQFHIIVCGLDSIIAR

S cerevisiae/1-206      RFINETLVKLTLES-----NYEICIPFIDGGTEGLKGHVKTIIPGITACWECSIDTLPSQ
S pombe/1-268           RWINSTLVAIAK--------TGDLIPLVDGGSEGLKGQARVIIPTITSCYECSLDMLTPK
Owl limpet/1-255        RWINGMLVSLVKYE-DGELDQSSLIPMVDGGTEGFKGNARVILPGLTPCIDCTLDLYPPQ
Sea squirt/1-262        RWINGMLISLLEYDEDGNLDPSSLIPLVDGGTEGFKGNARVILPGMNACMDCTLDLYPPQ
Roundworm/1-257         RWINGMLCDLVLEMADGKPDENTIIPMIDGGTEGFKGNARVIYPKFTACIDCTLDLYPPQ
Fruit fly/1-262         RWINGMLLSMLRYEEDGTIDTSSIVPMIDGGTEGFKGNARVILPGFTACIECTLDLFPPQ
Octopus/1-269           RWMNRMLISLLNYE-DGILDQASVIPMVDGGTEGFKGNARVIFPGMSACIECTLDFYPPQ
Lancelet/1-273          RWMNGMVLSLINYEEDGSVDQSSIIPLIDGGTEGFKGNARVILPGITACMDCTLELYPPQ
Lamprey/1-281           RWINGMLMSMLNFE-DGVLDPSTIIPMIDGGTEGFKGNARVVLPGMTACIECTLDLYPPQ
Gray bichir/1-290       RWINGMLVSLLNYE-DGALDPSSIIPLIDGGTEGFKGNARVILPGMTACIDCTLDLYPPQ
Elephant shark/1-283    RWINGMLISLLNFE-ESTLDPSSIIPLIDGGTEGFKGNSRVILPGMTACIECTLELYPPQ
Zebrafish/1-283         RWMNGMLLSLLIYE-DGVLDPSSIIPLIDGGTEGFKGNARVILPGMTACIDCTLELYPPQ
Frog/1-263              RWLNGMLMSLLNYE-DGVLQQSTVIPLIDGGTEGFKGNSRVILPGMTACVECTLELYPPQ
Mouse/1-263             RWINGMLISLLNYE-DGVLDPSSIVPLIDGGTEGFKGNARVILPGMTACIECTLELYPPQ
Human/1-263             RWINGMLISLLNYE-DGVLDPSSIVPLIDGGTEGFKGNARVILPGMTACIECTLELYPPQ

S cerevisiae/1-206      QDTVPMCTIANNPRCIEHVVEYVSTIQYPDLNIESTADM---------------------
S pombe/1-268           -ISYPICTLANTPRLPEHCVEWAYLLEWPRVFLNASVDSFSKQEVFEPLDGKNSNFEPDN
Owl limpet/1-255        -VNFPLCTIAHTPRLPEHCIEYIRILLWPKEEP----------------FGEGVIIDGDD
Sea squirt/1-262        -VNFPMCTIAHTPRQPEHCIEYVKIFQWEKEKP----------------FGD-IAIDGDD
Roundworm/1-257         -VNFPLCTIAHTPRLPEHCIEYIKVVVWPEEKP----------------FE-GVSLDADD
Fruit fly/1-262         -VNYPLCTIANTPRLPEHCIEYVKIIQWEKQNP----------------FG--VPLDGDD
Octopus/1-269           -INFPLCTIAHTPRLPEHCIEYVRLLLWPKQEP----------------FGANVAIDGDD
Lancelet/1-273          -VTYPMCTIAHTPRLPEHCIEWVRILLWPQEKP----------------FGEEVGIDGDD
Lamprey/1-281           -INFPICTLASMPRLPEHCIEYVRLLQWPKEQP----------------FGDAVALDGDD
Gray bichir/1-290       -INFPMCTIASMPRLPEHCIEYVRVLQWPKEQP----------------FGDGVTLDGDD
Elephant shark/1-283    -INFPMCTIASMPRLPEHCIEYARILQWPKENP----------------FGEGIALDGDD
Zebrafish/1-283         -INFPMCTIASMPRLPEHCVEYVRMLLWPKEKP----------------FGDGVVLDGDD
Frog/1-263              -INFPMCTIASMPRLPEHCIEYVRILQWPKEQP----------------FGEGVQLDGDD
Mouse/1-263             -VNFPMCTIASMPRLPEHCIEYVRMLQWPKEQP----------------FGDGVPLDGDD
Human/1-263             -VNFPMCTIASMPRLPEHCIEYVRMLQWPKEQP----------------FGEGVPLDGDD

S cerevisiae/1-206      ----EFLLEK
S pombe/1-268           IRHIDWLVKR
Owl limpet/1-255        PSHINWILKK
Sea squirt/1-262        PDHIKWICKA
Roundworm/1-257         PIHVEWVLER
Fruit fly/1-262         PQHIGWIYER
Octopus/1-269           PQHIKWIYEK
Lancelet/1-273          PAHIKWIYDR
Lamprey/1-281           PEHIQWVYQN
Gray bichir/1-290       PEHIQWVFHK
Elephant shark/1-283    PEHIQWVFQK
Zebrafish/1-283         PKHIQWVYQK
Frog/1-263              PEHIQWIFMN
Mouse/1-263             PEHIQWIFQK
Human/1-263             PEHIQWIFQK
```
