# Supplementary material for: A novel region within a conserved domain in ATG7 emerged in vertebrates
Source: Autophagy Rep. 2022 Sep 7;1(1):393–413. doi: 10.1080/27694127.2022.2118933 (PMC11864663; doi:10.1080/27694127.2022.2118933)
Supplement: Supplemental Material [file KAUO_A_2118933_SM3441.zip › FigureS3.pdf]

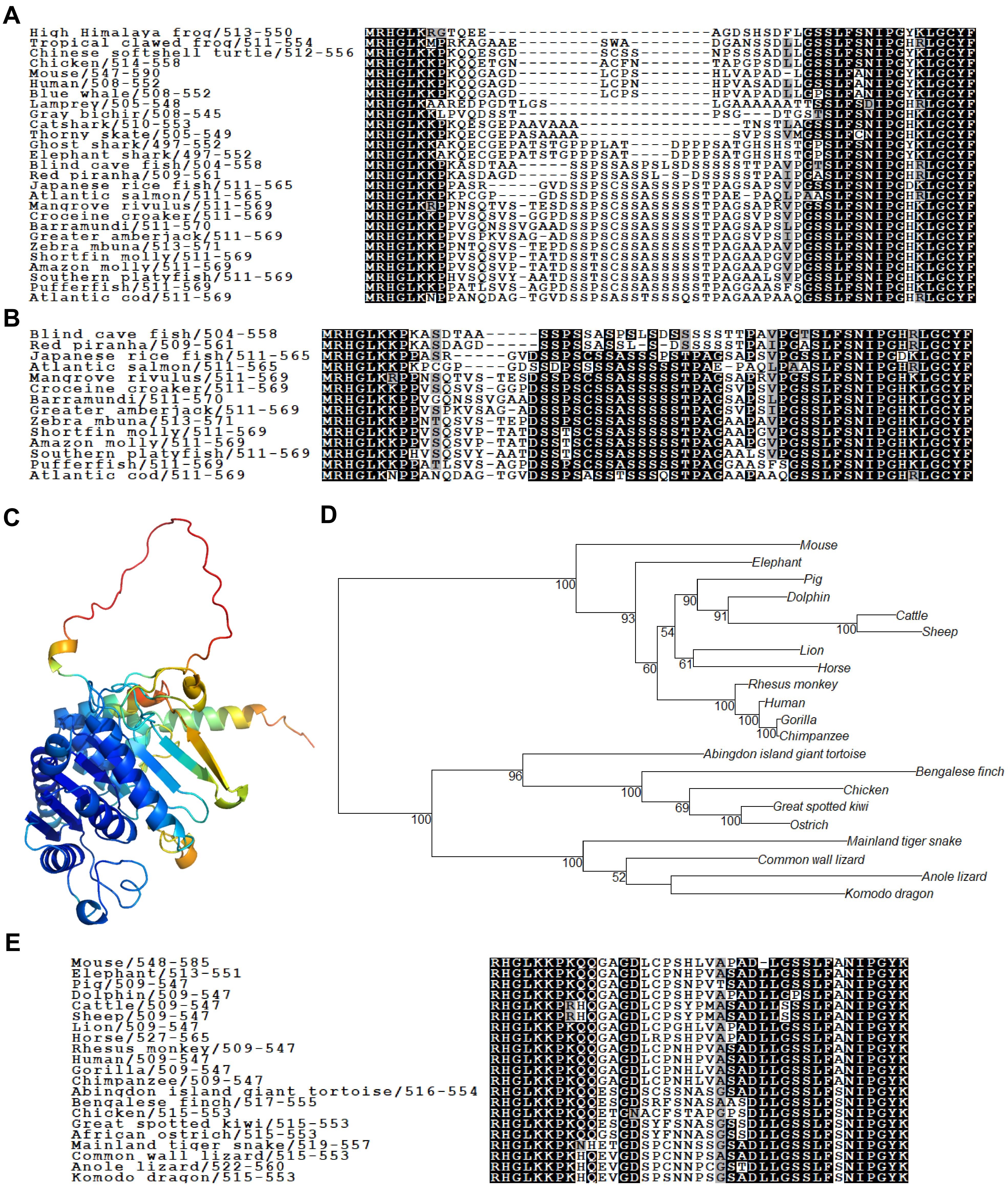
A
High Himalaya frog/513-550
Tropical clawed frog/511-554
Chinese softshell turtle/512-556
Chicken/514-558
Mouse/547-590
Human/508-552
Blue whale/508-552
Lamprey/505-548
Gray bichir/508-545
Catshark/510-553
Thorny skate/505-549
Ghost shark/497-552
Elephant shark/497-552
Blind cave fish/504-558
Red piranha/509-561
Japanese rice fish/511-565
Atlantic salmon/511-565
Mangrove rivulus/511-569
Croceine croaker/511-569
Barramundi/511-570
Greater amberjack/511-569
Zebra mbuna/513-571
Shortfin molly/511-569
Amazon molly/511-569
Southern platyfish/511-569
Pufferfish/511-569
Atlantic cod/511-569
B
Blind cave fish/504-558
Red piranha/509-561
Japanese rice fish/511-565
Atlantic salmon/511-565
Mangrove rivulus/511-569
Croceine croaker/511-569
Barramundi/511-570
Greater amberjack/511-569
Zebra mbuna/513-571
Shortfin molly/511-569
Amazon molly/511-569
Southern platyfish/511-569
Pufferfish/511-569
Atlantic cod/511-569
C
D
Mouse
Elephant
Pig
Dolphin
Cattle
Sheep
Lion
Horse
Rhesus monkey
Human
Gorilla
Chimpanzee
Abingdon island giant tortoise
Bengalese finch
Chicken
Great spotted kiwi
Ostrich
Mainland tiger snake
Common wall lizard
Anole lizard
Komodo dragon
100
93
90
91
100
54
61
60
100
100
100
96
100
69
100
100
100
52
E
Mouse/548-585
Elephant/513-551
Pig/509-547
Dolphin/509-547
Cattle/509-547
Sheep/509-547
Lion/509-547
Horse/527-565
Rhesus monkey/509-547
Human/509-547
Gorilla/509-547
Chimpanzee/509-547
Abingdon island giant tortoise/516-554
Bengalese finch/517-555
Chicken/515-553
Great spotted kiwi/515-553
African ostrich/515-553
Mainland tiger snake/519-557
Common wall lizard/515-553
Anole lizard/522-560
Komodo dragon/515-553
