# Supplementary material for: A novel region within a conserved domain in ATG7 emerged in vertebrates
Source: Autophagy Rep. 2022 Sep 7;1(1):393–413. doi: 10.1080/27694127.2022.2118933 (PMC11864663; doi:10.1080/27694127.2022.2118933)
Supplement: Supplemental Material [file KAUO_A_2118933_SM3441.zip › FigureS4.pdf]

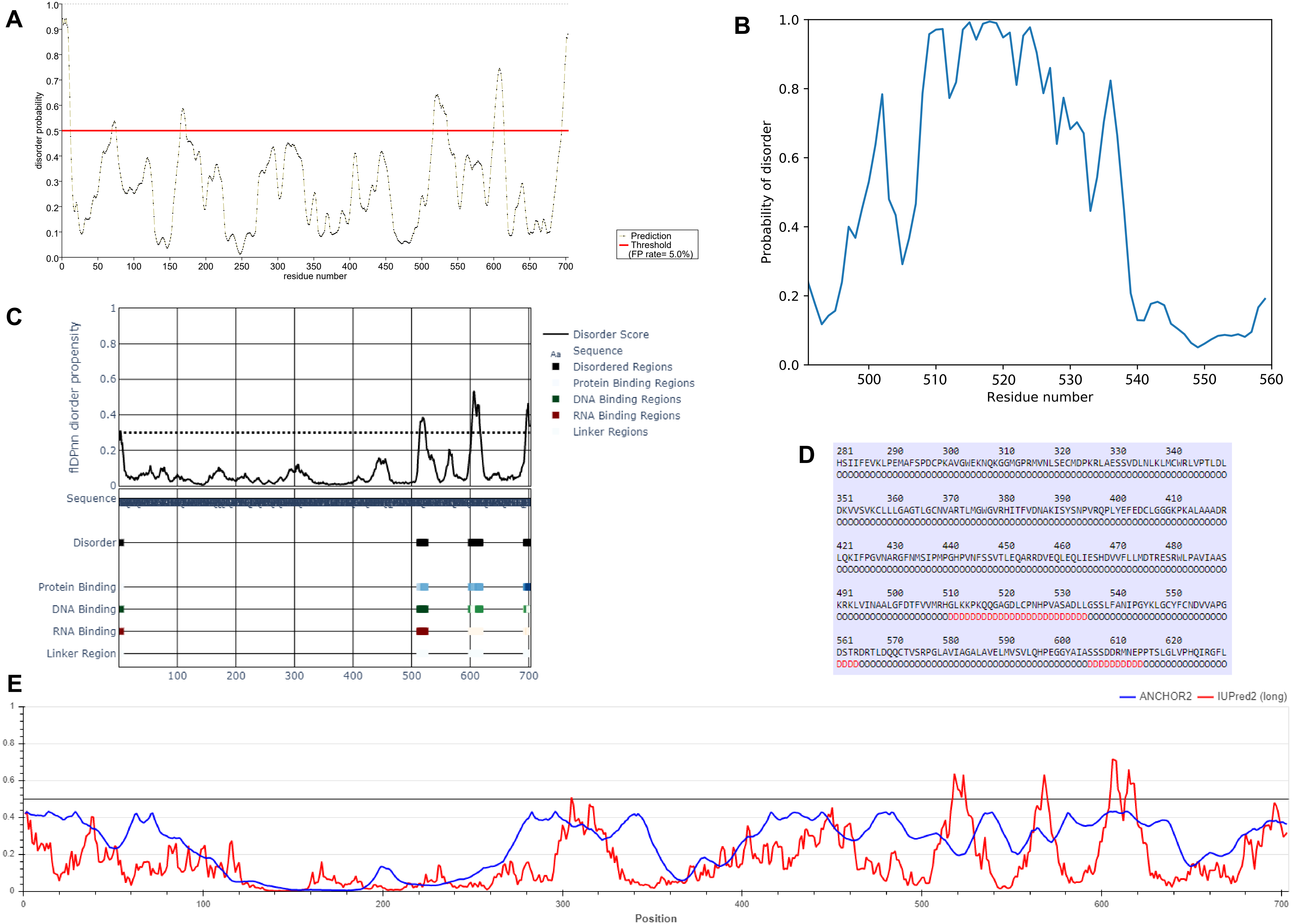

A
disorder probability
residue number
Prediction
Threshold
(FP rate= 5.0%)
B
Probability of disorder
Residue number
C
fIDPnn disorder propensity
Disorder Score
Sequence
Disordered Regions
Protein Binding Regions
DNA Binding Regions
RNA Binding Regions
Linker Regions
Sequence
Disorder
Protein Binding
DNA Binding
RNA Binding
Linker Region
D
281 290 300 310 320 330 340
HSIIFEVKLPEMAFSPDCPKAVGWEKNQKGGMGPRMVNLSECMDPKRLAESSVDLNLKLMCWRLVPTLDL
OOOOOOOOOOOOOOOOOOOOOOOOOOOOOOOOOOOOOOOOOOOOOOOOOOOOOOOOOOOOOOOOOOOOOO
351 360 370 380 390 400 410
DKVVSVKCLLLGAGTLGCNVARTLMGWGVRHITFVDNAKISYSNPVRQPLYEFEDCLGGGKPKALAAADR
OOOOOOOOOOOOOOOOOOOOOOOOOOOOOOOOOOOOOOOOOOOOOOOOOOOOOOOOOOOOOOOOOOOOOO
421 430 440 450 460 470 480
LQKIFPGVNARGFNMSIPMPGHPVNFSSVTLEQARRDVEQLEQLIESHDVVFLLMDTRESRWLPAVIAAS
OOOOOOOOOOOOOOOOOOOOOOOOOOOOOOOOOOOOOOOOOOOOOOOOOOOOOOOOOOOOOOOOOOOOOO
491 500 510 520 530 540 550
KRKLVINAALGFDTFVVMRHGLKKPKQQGAGDLCPNHPVASADLLGSSLFANIPGYKLGCYFCNDVVAPG
OOOOOOOOOOOOOOOOOOOODDDDDDDDDDDDDDDDDDDDDDDDDOOOOOOOOOOOOOOOOOOOOOOOOO
561 570 580 590 600 610 620
DSTRDRTLDQQCTVSRPGLAVIAGALAVELMVSVLQHPEGGYAIASSSDDRMNEPPTSLGLVPHQIRGFL
DDDDOOOOOOOOOOOOOOOOOOOOOOOOOOOOOOOOOOOODDDDDDDDDDOOOOOOOOOOOOOOOOOOOO
E
ANCHOR2
IUPred2 (long)
Score
Position
